# The Relationship Between Caregiver Burden and Posttraumatic Growth in Caregivers of Patients With Metastatic Cancer

**DOI:** 10.7759/cureus.23622

**Published:** 2022-03-29

**Authors:** Pınar Eraslan, Aysegul İlhan, Emrah Eraslan, Cengiz Karacin, Ömür Berna Çakmak Öksüzoğlu

**Affiliations:** 1 Psychiatry, Presidential Health Center, Ankara, TUR; 2 Medical Oncology, University of Health Sciences Dr Abdurrahman Yurtarslan Ankara Oncology Training and Research Hospital, Ankara, TUR

**Keywords:** family, psychosocial problems, metastatic cancer, depression, post-traumatic growth, caregiver burden

## Abstract

Objective

In this study, we aimed to examine the effect of post-traumatic growth and depressive symptoms on caregiver burden in caregivers of cancer patients.

Methods

This was a single-center cross-sectional observational descriptive study conducted at a medical oncology clinic. The study included 214 caregivers of cancer patients. Participants were assessed with a sociodemographic information form, the Turkish versions of the Zarit Caregiver Burden Scale (ZCBS), the Posttraumatic Growth Inventory (PTGI), and the Beck Depression Inventory (BDI).

Results

The mean ZCBS, PTGI, and BDI scores were 42.7 ±13.8, 67.8 ±22.3, and 13.5 ±9.8, respectively. There was a negative correlation (r=-0.407, p<0.001) between the ZCBS and the PTGI total scores, a positive correlation (r=0.636, p<0.001) between the ZCBS total and BDI scores, and a negative correlation (r=-0.426, p<0.001) between the PTGI total and BDI scores. Age, gender, income level, and history of psychiatric treatment were not independent predictive factors for the ZCBS total scores. PTGI total score (B=-0.107, 95% CI: -0.178 to -0.037, p=0.003) and BDI score (B=0.776, 95% CI: 0.602-0.950, p<0.001) were independent predictive factors for ZCBS total scores.

Conclusions

Our study revealed a significant negative relationship between caregiver burden and PTGI in caregivers of metastatic cancer patients, and it was found that depression negatively affects burden in caregivers. Posttraumatic growth can be a protective buffer against the burden of care and depression among caregivers.

## Introduction

Cancer is a condition that causes many physical, mental, and psychosocial problems for patients and their relatives, from diagnosis to treatment, including death and mourning [1]. It significantly affects the family's economic and psychosocial life level as it leaves an impact on the whole family by causing psychosocial trauma to the patient and their relatives [2].

The concept of “posttraumatic growth” (PTG) put forward by Tedeschi and Colhoun posits that the individual experiences a sense of growth that goes beyond their previous level of functionality and awareness as a result of the trauma [3]. The concept of PTG is used to describe the positive psychological changes, as well as cognitive, emotional, and behavioral transformations that develop due to a traumatic event [4]. Individuals who experience PTG can better understand the meaning of life, improve their interpersonal relationships, improve themselves spiritually, and realize new possibilities by being aware of themselves and their environment [4]. Some studies have shown a positive relationship between PTG and resilience [5]. This, in turn, results in increased social performance and the ability to overcome problems after exposure to severe stress and risk factors. While studies on PTG in cancer are mostly related to cancer patients [6,7], the number of studies on PTG in the relatives and caregivers of cancer patients has been on the rise in recent years [8,9].

Advances in cancer treatment have enabled the shifting of patient care to the home environment and enabled family members to assume significant roles as caregivers [10]. Family caregivers are required to undertake many tasks, including disease and treatment monitoring, symptom management, medication management, emotional and financial support, and personal care [11]. Caregiving burden is defined as the distress that caregivers feel as a result of caregiving [11]. Helping cancer patients cope with their cancer-related emotions and providing emotional support to them is a psychologically challenging task for caregivers [12]. Being unable to work and neglecting social relations due to caregiving responsibility can be considered a social burden [12]. The economic burden entails paying high medical expenses and losing income and savings [12].

While a cancer diagnosis in an individual in a family can cause trauma for the fellow family members, it can bring positive changes in the family as well [8]. In the study by Cormio et al. investigating psychological well-being and posttraumatic growth in caregivers, “personal strength” was evaluated as a positive effect in caregivers of cancer patients [8]. We hypothesized that this outcome might be an influential factor on the caregiver burden. The findings of the only study that investigated the relationship between caregiver burden and PTG in the literature suggest that caregivers may experience burden when caring for a relative with cancer [1]. However, caregivers can also experience growth, especially in the way they relate with others and appreciate life [1]. In our study, we aimed to contribute to the literature on this subject by exploring the relationship between care burden and PTG in caregivers of metastatic cancer patients in our country.

## Materials and methods

This was a single-center cross-sectional observational descriptive study carried out at the medical oncology department of a tertiary referral center between January 2020 and March 2021. The local ethics committee approval (University of Health Sciences Dr Abdurrahman Yurtarslan Ankara Oncology Training and Research Hospital Clinical Research Ethics Committee, Approval Date: 04/12/2019, Document No: 2019-12/474) was obtained before the start of the study.

Study population

Relatives/caregivers of patients diagnosed with metastatic cancer were included in the study. We defined the term caregiver as a person who primarily supports the patient in meeting the daily basic life needs and is the decision-maker together with the patient in matters related to the patient's treatment. All participants were over 18 years of age, literate, and without any physical or mental disabilities. Participants with severe and uncontrolled comorbidities (i.e., heart failure, chronic obstructive pulmonary disease, neurological disease, liver failure, or renal failure) were excluded, while participants with non-life-threatening and well-controlled comorbidities were allowed to enroll in the study; 214 participants who met the study criteria and signed the informed consent form were included in the study. A printed sociodemographic information form, Zarit Caregiver Burden Scale (ZCBS), Posttraumatic Growth Inventory (PTGI), and Beck Depression Inventory (BDI) were provided to the participants. The participants were asked to fill out the questionnaires themselves in a quiet environment.

Instruments

Demographic and Medical Information Form 

A pre-designed demographic information form was distributed among the participants, which included questions about age, marital status (single or married), comorbidities, educational status, income level (to be reported as low, medium, or high based on their own opinions), and history of psychiatric treatment (current or past).

Zarit Caregiver Burden Scale (ZCBS)

The Zarit Caregiver Burden Scale is a Likert-type scale consisting of 22 questions with scores ranging between 0 and 5; it is used to evaluate the stress experienced by caregivers of individuals in need of care [13]. Based on the study conducted about the validity and reliability of the ZCBS in the Turkish context among caregivers of patients with schizophrenia, Cronbach's alpha coefficient was 0.83, and three items were removed from the scale [14]. The five sub-dimensions in the tool were defined as follows: 1: mental strains and impaired private life, 2: nervousness and restrictedness, 3: impaired social relationships, 4: financial burden, and 5: dependency [13]. The score can be obtained from the scale, which varies between 19 and 95 points, and a high score indicates a high caregiver burden.

Posttraumatic Growth Scale (PTGI)

The Posttraumatic Growth Inventory is a 21-item Likert-type scale with scores ranging between 0-5 [4]. The total score of the scale is in the range of 0-105. A high score indicates that the person has experienced a high level of growth after the traumatic experience. Five sub-dimensions were identified: relationships with others, new possibilities, personal strength, spiritual change, and appreciation for life. Işıklı and Dürü determined the validity and reliability of the Turkish version of the PTGI with a Cronbach's alpha coefficient of 0.93 [15].

Beck Depression Inventory (BDI)

The Beck Depression Inventory consists of 21 questions, each of which has a score between 0 and 3 [16]. High scores on this scale indicate an increase in the severity of depressive complaints. In the study by Hisli et al. that evaluated the validity and reliability of the Turkish version of the BDI, it was observed that a scale score of 17 and above indicated that the level of depression was above normal [17].

Statistical analysis

Statistical analysis was performed using the SPSS Statistics software for Windows, version 24.0 (IBM, Armonk, NY). The Kolmogorov-Smirnov test was used to test the compatibility of the data to a normal distribution. Since the number of study participants was more than 200, numerical data were presented in the form of means and standard deviations, parametric tests were used, and a linear regression model was carried out [18]. Categorical data were presented as frequencies and percentages. In order to determine the difference between the mean scores of the study scales in the groups formed according to demographic characteristics, the Student's t-test was used for the comparison of subgroups, and the one-way analysis of variance (ANOVA) test was used if there were more than two subgroups. Pearson correlation analysis including research scale total scores and subscale scores was performed. Multivariate linear regression analysis was performed using variables with a p-value below 0.05 as per the univariate analysis to determine independent factors predicting ZCBS total score. All statistical tests were two-sided, and a p-value <0.05 was considered statistically significant.

## Results

The study included 214 caregivers of metastatic cancer patients, with a mean age of 42.8 ±12.5 years. Of the participants, 110 (51.4%) were female, and 104 (48.6%) were male. The caregivers of the patients mainly comprised their children (n=134, 62.6%). The mean ZCBS, PTGI, and BDI scores were 42.7 ±13.8, 67.8 ±22.3, and 13.5 ±9.8, respectively. The main sociodemographic characteristics of the participants and the study scale scores are shown in Table 1.

**Table 1:** Main sociodemographic characteristics of the participants and the study scale scores ^§^According to the participants’ statements SD: Standard deviation; ZCBS: Zarit Caregiver Burden Scale; PTGI: Posttraumatic Growth Inventory; BDI: Beck Depression Inventory

Parameters		N	%
Age in years, mean ±SD		42.8 ±12.5	
Gender			
	Female	110	51.4
	Male	104	48.6
Marital status			
	Single	53	24.8
	Married	161	75.2
Educational status			
	Primary school	81	37.9
	High school	58	27.1
	University	75	35.0
Employment status			
	No	118	55.1
	Yes	96	44.9
Income status^§^			
	Low	79	36.9
	Moderate and high	135	63.1
Place of residence			
	Rural	58	27.1
	Urban	156	72.9
Comorbidity			
	No	188	87.9
	Yes	26	12.1
Psychiatric treatment history			
	No	188	87.9
	Yes	26	12.1
Degree of kinship with the patient			
	Spouse	68	31.8
	Child	134	62.6
	Brother	12	5.6
ZCBS score, mean ±SD			
	Total	42.7 ±13.8	
	ZCBS1	12.0 ±5.9	
	ZCBS2	5.9 ±2.8	
	ZCBS3	4.9 ±2.3	
	ZCBS4	12.8 ±3.9	
	ZCBS5	6.0 ±2.7	
PTGI score, mean ±SD			
	Total	67.8 ±22.3	
	PTGI1	21.0 ±8.8	
	PTGI2	13.6 ±6.4	
	PTGI3	14.5 ±4.6	
	PTGI4	8.4 ±2.4	
	PTGI5	10.7 ±3.7	
BDI score, mean ±SD		13.5 ±9.8	

Mean scores obtained from the study scales according to the sociodemographic characteristics of the participants were compared. Mean ZCBS scores of participants with low-income levels were higher than those with moderate-/high-income levels (45.9 ±12.4 and 40.9 ±14.3, p=0.009), and mean scores of those with a history of psychiatric treatment were higher than those without a history of psychiatric treatment (49.1 ±13.7 and 41.9 ±13.6, p=0.012). Mean PTGI scores were similar as per the comparison based on the sociodemographic characteristics of the participants. Mean BDI scores of married participants were higher than those of singles (14.5 ±10.4 and 10.6 ±6.9, p=0.002); mean scores of primary school graduates were higher than high school/university graduates (16.0 ±11.2 and 12.2 ±8.9/11.8 ±8.3, p=0.013); mean scores of those with low-income levels were higher than those with middle/high income (16.4 ±10.6 and 11.8 ±8.7, p=0.001); mean scores of those with a psychiatric treatment history were higher than those with no psychiatric treatment history (21.2 ±9.5 and 12.5 ±9.4, p<0.001); and mean scores of spouses of the patients were higher than children/siblings of the patients (16.3 ±12.1 and 12.5 ±8.0/9.0 ±10.2, p=0.008). The study scale scores according to the sociodemographic characteristics of the participants are shown in Table 2.

**Table 2:** Study scale scores according to the sociodemographic characteristics of the participants ^§^According to the participants' statements SD: standard deviation; ZCBS: Zarit Caregiver Burden Scale; PTGI: Posttraumatic Growth Inventory; BDI: Beck Depression Inventory

Parameters		ZCBS, mean ±SD	P-value	PTGI, mean ±SD	P-value	BDI, mean ±SD	P-value
Gender			0.389		0.898		0.201
	Female	41.9 ±14.2		68.0 ±19.1		12.7 ±9.3	
	Male	43.6 ±13.4		67.6 ±25.4		14.4 ±10.3	
Marital status			0.259		0.744		0.002
	Single	40.9 ±13.1		67.0 ±19.1		10.6 ±6.9	
	Married	43.3 ±14.0		68.1 ±23.3		14.5 ±10.4	
Educational status			0.675		0.181		0.013
	Primary school	43.4 ±13.5		67.4 ±22.6		16.0 ±11.2	
	High school	41.4 ±12.4		72.1 ±23.2		12.2 ±8.9	
	University	43.0 ±15.3		64.9 ±21.1		11.8 ±8.3	
Employment status			0.858		0.264		0.847
	No	42.6 ±14.4		69.4 ±20.1		13.4 ±9.5	
	Yes	42.9 ±13.1		65.9 ±24.8		13.7 ±10.2	
Income status^§^			0.009		0.928		0.001
	Low	45.9 ±12.4		67.6 ±20.8		16.4 ±10.6	
	Moderate and high	40.9 ±14.3		67.9 ±23.3		11.8 ±8.7	
Place of residence			0.445		0.284		0.069
	Rural	43.9 ±13.5		65.1 ±24.2		15.5 ±10.6	
	Urban	42.3 ±14.0		68.8 ±21.6		12.8 ±9.4	
Comorbidity			0.372		0.068		0.190
	No	42.4 ±14.1		66.8 ±22.5		13.2 ±9.9	
	Yes	45.0 ±11.0		75.3 ±19.7		15.9 ±8.6	
Psychiatric treatment history			0.012		0.055		<0.001
	No	41.9 ±13.6		68.9 ±22.2		12.5 ±9.4	
	Yes	49.1 ±13.7		59.9 ±21.8		21.2 ±9.5	
Degree of kinship with the patient			0.380		0.463		0.008
	Spouse	44.2 ±13.5		68.2 ±23.2		16.3 ±12.1	
	Child	42.4 ±13.9		68.3 ±22.2		12.5 ±8.0	
	Sibling	38.5 ±14.4		60.0 ±18.9		9.0 ±10.2	

The results of the correlation analysis, including study scale total scores and subscale scores, are shown in Table 3. There was a moderate negative correlation (r=-0.407, p<0.001) between the ZCBS and the PTGI total scores. There was a strong positive correlation (r=0.636, p<0.001) between the ZCBS total and BDI scores. There was a moderate negative correlation (r=-0.426, p<0.001) between the PTGI total and BDI scores.

**Table 3:** Correlation analysis results including study scale total scores and subscale scores *p<0.05; **p<0.01 ZCBS: Zarit Caregiver Burden Scale; PTGI: Posttraumatic Growth Inventory; BDI: Beck Depression Inventory

		1	2	3	4	5	6	7	8	9	10	11	12	13
1. ZCBS1	r	1												
	p													
2. ZCBS2	r	0.701^**^	1											
	p	<0.001												
3. ZCBS3	r	0.709^**^	0.603^**^	1										
	p	<0.001	<0.001											
4. ZCBS4	r	0.458^**^	0.365^**^	0.341^**^	1									
	p	<0.001	<0.001	<0.001										
5. ZCBS5	r	0.347^**^	0.445^**^	0.235^**^	0.472^**^	1								
	p	<0.001	<0.001	0.001	<0.001									
6. ZCBS total	r	0.898^**^	0.799^**^	0.743^**^	0.690^**^	0.583^**^	1							
	p	<0.001	<0.001	<0.001	<0.001	<0.001								
7. PTGI1	r	-0.466^**^	-0.350^**^	-0.330^**^	-0.147^*^	-0.087	-0.389^**^	1						
	p	<0.001	<0.001	<0.001	0.032	0.206	<0.001							
8. PTGI2	r	-0.434^**^	-0.306^**^	-0.344^**^	-0.131	-0.108	-0.377^**^	0.786^**^	1					
	p	<0.001	<0.001	<0.001	0.056	0.116	<0.001	<0.001						
9. PTGI3	r	-0.421^**^	-0.336^**^	-0.341^**^	-0.100	-0.134	-0.370^**^	0.789^**^	0.749^**^	1				
	p	<0.001	<0.001	<0.001	0.144	0.051	<0.001	<0.001	<0.001					
10. PTGI4	r	-0.173^*^	-0.114	0.016	0.041	-0.007	-0.089	0.431^**^	0.433^**^	0.489^**^	1			
	p	0.011	0.097	0.813	0.550	0.915	0.194	<0.001	<0.001	<0.001				
11. PTGI5	r	-0.363^**^	-0.309^**^	-0.158^*^	-0.181^**^	-0.097	-0.324^**^	0.671^**^	0.660^**^	0.566^**^	0.487^**^	1		
	p	<0.001	<0.001	0.021	0.008	0.156	<0.001	<0.001	<0.001	<0.001	<0.001			
12. PTGI total	r	-0.478^**^	-0.365^**^	-0.336^**^	-0.141^*^	-0.114	-0.407^**^	0.943^**^	0.903^**^	0.876^**^	0.555^**^	0.780^**^	1	
	p	<0.001	<0.001	<0.001	0.039	0.096	<0.001	<0.001	<0.001	<0.001	<0.001	<0.001		
13. BDI	r	0.554^**^	0.563^**^	0.431^**^	0.537^**^	0.289^**^	0.636^**^	-0.399^**^	-0.396^**^	-0.287^**^	-0.134	-0.486^**^	-0.426^**^	1
	p	<0.001	<0.001	<0.001	<0.001	<0.001	<0.001	<0.001	<0.001	<0.001	0.050	<0.001	<0.001	

The results of the multivariate linear regression analysis, in which the factors with a p-value less than 0.05 were included in the univariate linear regression analysis, which included the factors that could predict the ZCBS total score, are shown in Table 4. Age, gender, income level, and history of psychiatric treatment were not independent predictive factors for the ZCBS total score. PTGI total score (B=-0.107, 95% CI: -0.178 to -0.037, p=0.003) and BDI score (B=0.776, 95% CI: 0.602-0.950, p<0.001) were independent predictive factors for ZCBS total score.

**Table 4:** Results of multivariate linear regression analysis including factors that may predict ZCBS total score Note: R2adj=0.418 (n=214, p<0.001) CI: confidence interval for B; ZCBS: Zarit Caregiver Burden Scale; PTGI: Posttraumatic Growth Inventory; BDI: Beck Depression Inventory

Parameters	B	95% CI		β	t	p
		Lower	Upper			
Age, years	0.083	-0.034	0.199	0.075	1.397	0.164
Gender (male vs. female)	-0.191	-3.114	2.731	-0.007	-0.129	0.897
Income status (moderate/high vs. low)	-1.073	-4.140	1.994	-0.038	-0.690	0.491
Psychiatric treatment (yes vs. no)	-0.893	-5.524	3.737	-0.021	-0.380	0.704
PTGI total score	-0.107	-0.178	-0.037	-0.174	-2.990	0.003
BDI score	0.776	0.602	0.950	0.550	8.807	<0.001

## Discussion

The current study showed a negative and significant relationship between the ZCBS and PTGI total scores among caregivers of patients with metastatic cancer. Furthermore, a positive correlation between the BDI and ZCBS total scores and a negative correlation between the BDI and PTGI total scores were observed. Also, the PTGI and BDI total scores independently predicted the ZCBS total scores while other factors such as age, gender, income level, and history of psychiatric treatment did not.

Although cancer is a traumatic experience for both patients and their families, positive outcomes may emerge by channeling the pain from such a traumatic experience into positive, productive, and meaningful growth through finding meaning in the experience [19]. Cancer may produce profound changes in the whole family system, and both the patient and his/her caregiver may experience growth after the experience of illness [8,20]. Our study findings indicated that PTG might offer a protective barrier against the burden of care and depression in caregivers of metastatic cancer patients.

To the best of our knowledge, only one other study has assessed the relationship between caregiver burden and PTG in caregivers of cancer patients apart from our study. Teixeira et al. in their study involving 74% of females among 214 adult children of patients undergoing chemotherapy participants observed that caregivers could experience burden and growth together while caring for a relative with cancer [1]. Moreover, higher burden and posttraumatic growth were observed in female caregivers whose parents had been ill for less than a year and who perceived their parents as completely dependent on them [1]. In our study, unlike the results of this study, caregivers' growth was associated with lower levels of care burden. The difference between the two study findings can be explained in several ways. Firstly, it entails the idea that fulfilling the role of the caregiver, with the contribution of cultural and religious factors, can give the individual a sense of fulfillment, make him/her feel competent and able to cope with difficulties, and consequently reduces the sense of burden related to caregiving. On the other hand, the experience of caregiving may activate the internal resources of the individual. Thus, positive outcomes can occur by channeling the pain from such traumatic experiences into positive and meaningful growth by finding meaning in the experience. In addition, the family structure (close family ties and social relations) of the Turkish society may play a positive role in equipping family members to accept traumatic diseases such as cancer with sympathy and compassion, cope with the disease with fortitude, accept the burden of care more humanely, and gain maturity after traumatic experiences. In addition, unlike the study of Teixeira et al., the fact that 51.4% of caregivers in the sample group were female in our study, and that spouses and siblings were included in addition to adult children, may explain the difference in the findings.

Another study involving daughters of breast cancer survivors found that higher PTGI scores and PTG were associated with enhanced social support (SS), emotional processing strategies, and problem-focused coping strategies compared to women with healthy parents [21]. Women who use more adaptive coping strategies such as planning, active coping, seeking SS, and processing emotions report higher PTG [21]. Tedeschi and Calhoun suggested that some individual and contextual factors such as coping strategies, individual schemata [22], and SS facilitate the development of PTG. Also, longer disease duration and prolonged treatments were both associated with more growth [1]. We observed in our previous study, which evaluated the coping strategies in mothers of children with congenital heart disease, that the most prominent coping strategy was problem-focused coping strategies and associated with lower levels of depression [23]. Similarly, the studies of Thompson et al. and Rao et al. have revealed that women using problem-focused coping strategies had lower depression scores [24,25]. Accordingly, we hypothesized that adaptive coping strategies may be associated with a lower burden and higher PTG as protective factors against depression, and this may form the underlying idea of our current study findings.

In the present study, we found a significant negative relationship between the BDI and PTGI scores. In a study by Nouzari et al., which evaluated the relationship between PTG and SS as well as hope in caregivers of gastrointestinal cancer patients, a significant positive correlation was found between PTG and both SS (p<0.001, r=0.59) and hope (p<0.001, r=0.70) [26]. The existence of a correlation between PTG and hope in this study coincides with the negative correlation between PTGI and BDI scores in our study. However, the data in the litrature is confusing because of the conflict between study findings stating that there is a positive correlation [27] between PTG and depression and those reporting that there is no relationship [8]. In our study, we assumed that effective coping methods might have facilitated higher PTG and thus contributed positively to the mental state of caregivers with fewer depression levels. In the only study investigating the relationship between burden and PTG in caregivers in diseases other than cancer, involving the relatives of caregivers of schizophrenia patients, no significant difference was found between caregivers with high and low PTG in terms of burden, burnout, and SS [14]. The power of this study may have been insufficient to show the relationship between PTG and other parameters due to the small sample size [14]. However, the existence of such a study can endorse the idea that there may be a connection between PTG, burnout, and SS parameters. There is a need for large-scale and well-designed studies to evaluate the relationship between PTG and psychiatric conditions as there is limited data in the current literature characterized by conflicting findings from various studies.

Consistent with the data in the literature, we also observed a significant positive correlation between BDI and ZCBS total scores [28,29]. The study by Oven et al., involving 302 cancer patients and their caregivers to determine the predictive factors of caregiver burden, found that depression was positively correlated (p<0.001, r ¼ 0.381) with caregiver burden and the presence of depression was an independent predictor of a higher burden for caregivers [2]. Seo et al. have shown that the most prominent factor affecting the burden of care was depression among caregivers of patients with lung cancer [30]. These study findings reveal a close clinical relationship between depression and caregiving burden.

Study limitations

One of the limitations of our study is that the participants were recruited from a single oncology center that included different cancer patients, and we did not analyze the performance status of the patients, which could also affect the caregiver burden and PTG. However, since the hospital where the study was conducted is a large oncology clinic that caters to patients from all over Turkey, this study's findings may legitimately claim to be representative of the broader Turkish population. Another limitation of our study was relying on self-reported scales, which could negatively affect the responses to the questionnaires. For example, the use of the Hamilton Depression Rating Scale (HDRS), which could be more objective than BDI, would have given more significant findings in terms of depression. However, we used a high value of 17, which Hisli et al. determined as the clinically significant BDI cut-off score for depression [17]. In this way, we believe we have almost accurately identified a high-risk group for depression. Despite its limitations, our study may be important since it was conducted in a research area with limited and contradictory literature data, and it can form a basis for the design of new studies. Based on the findings of our study, further prospective, longitudinal intervention studies targeting PTG in cancer patient caregivers can be designed. These studies can contribute to the efforts to find solutions to the unfavorable psychiatric conditions among cancer caregivers such as depression, anxiety, illness, and fear of death, and hence the current gaps in this field in the literature can be filled.

## Conclusions

Our findings indicated a significant negative relationship between ZCBS and PTGI scores and a negative relationship between PTGI and BDI scores in caregivers of metastatic cancer patients. PTG can be considered a common influencing factor in caregiver burden and depression. Targeting PTG may positively contribute to addressing caregivers' burden and depression, and further intervention trials on PTG are needed. In this context, the effects of personality traits and coping strategies on PTG may be another potential area of research. Our study can be considered a starting point that opens the doors to this new area. Also, trials investigating the effects of psychological interventions to modify personality traits and coping strategies of cancer patient caregivers can provide clinical benefits in addressing the issues of PTG, depression, and caregiver burden.
